# NATURE EXPOSURE IS ASSOCIATED WITH POSITIVE VIEWS ON AGING

**DOI:** 10.1093/geroni/igae098.0857

**Published:** 2024-12-31

**Authors:** Jana Nikitin, Fiona Rupprecht, Christina Ristl, Selma Korlat

**Affiliations:** University of Vienna, Vienna, Wien, Austria; University of Vienna, Vienna, Wien, Austria; University of Vienna, Vienna, Wien, Austria; University of Vienna, Vienna, Wien, Austria

## Abstract

Could nature help us perceive the aging process more positively? We hypothesized that time spent in nature is associated with more positive views on aging (i.e., perceiving aging as more of a gain and less of a loss) because nature improves health and well-being and gives meaning in life. Study 1 (N = 289, age = 40-91 years) and Study 2 (N = 521, age = 40-88 years) were correlational studies examining self-reported frequency of spending time in nature in middle-aged and older adults. Both studies showed that spending more time in nature is associated with more positive views on aging. This relationship was mediated by subjective health, subjective well-being (Study 1) and meaning in life (Study 2), even after controlling for socioeconomic status. In Study 3, a field study (N = 338, age = 18-87 years), we surveyed people who were currently in a more or less natural environment (green spaces outside the city vs. city parks). We found that older (but not younger) participants in the more natural environment reported more positive attitudes on aging. This relationship was partially mediated by high levels of current subjective well-being and persisted after controlling for current physical activity and social context. In summary, contact with nature is of great benefit to us all, but particularly to older people when it comes to their attitudes to aging. Therefore, ensuring access to green spaces for older people is crucial and could serve as an intervention to promote positive experiences in old age.

